# Prevalence and heritability of handedness in a Hong Kong Chinese twin and singleton sample

**DOI:** 10.1186/s40359-020-00401-9

**Published:** 2020-04-22

**Authors:** Mo Zheng, Catherine McBride, Connie Suk-Han Ho, Jonathan Ka-Chun Chan, Kwong Wai Choy, Silvia Paracchini

**Affiliations:** 1 Department of Psychology, The Chinese University of Hong Kong, Hong Kong, China; 2 Department of Psychology, The University of Hong Kong, Hong Kong, China; 3 Department of Obstetrics & Gynaecology, The Chinese University of Hong Kong, Hong Kong, China; 4 School of Medicine, University of St Andrews, St. Andrews, Scotland

**Keywords:** Handedness, Edinburgh handedness inventory, Pegboard, Chinese children, Twins

## Abstract

**Background:**

Left-handedness prevalence has been consistently reported at around 10% with heritability estimates at around 25%. Higher left-handedness prevalence has been reported in males and in twins. Lower prevalence has been reported in Asia, but it remains unclear whether this is due to biological or cultural factors. Most studies are based on samples with European ethnicities and using the preferred hand for writing as key assessment. Here, we investigated handedness in a sample of Chinese school children in Hong Kong, including 426 singletons and 205 pairs of twins, using both the Edinburgh Handedness Inventory and Pegboard Task.

**Results:**

Based on a binary definition of writing hand, we found a higher prevalence of left-handedness (8%) than what was previously reported in Asian datasets. We found no evidence of increased left-handedness in twins, but our results were in line with previous findings showing that males have a higher tendency to be left-handed than females. Heritability was similar for both hand preference (21%) and laterality indexes (22%). However, these two handedness measures present only a moderate correlation (.42) and appear to be underpinned by different genetic factors.

**Conclusion:**

In summary, we report new reference data for an ethnic group usually underrepresented in the literature. Our heritability analysis supports the idea that different measures will capture different components of handedness and, as a consequence, datasets assessed with heterogeneous criteria are not easily combined or compared.

## Background

Handedness, the dominance of one hand over the other, is a commonly observed bias in human behavior and is probably the most studied human asymmetry. Handedness can be determined by measures of *preference* or *performance*. Preference refers to the preferred hand, left or right, used for daily tasks, whereas performance refers to the relative proficiency of one hand compared to the other for a specific manual task.

The most frequently used preference measure is the hand used for writing, which typically classifies people as left- or right-handed writers. More rarely, individuals with no clear preference are classified as ambidextrous. Hand preference is also assessed on the basis of a range of daily activities usually measured through standard questionnaire that collect self-reported answers or observations, such as the Annett Hand Preference Questionnaire [1], the Edinburgh Handedness Inventory [2], and the Waterloo Handedness Questionnaire [3]. Participants indicate their hand preference to each listed task/item, and a composite score is derived by combining all items. This composite score typically has a J-shaped distribution with most people preferring one hand over the other for most activities and the rest falling in different combinations in the middle. Using cutoff points on the preference composite score, people are classified into discrete handedness groups commonly represented by left-, mixed-, and right-handers. Those who fall in the middle category are referred to as mixed-handers, and they are those who prefer to use different hands for different tasks. Depending on the cutoff values used, the proportion of the three handedness groups can vary greatly [4–6].

Handedness performance is measured by comparing the relative skill of two hands on a specifically designed manual task. Some well-established performance measures include Annett’s peg moving task [1], Peters and Durding’s finger-tapping task [7] and Tapley and Bryden’s dot-filling task [8]. From these tasks, laterality indexes are derived by computing the difference of the two hands. An index will capture different dimensions of handedness which might not be detected by a categorical classification [9, 10]. Laterality indexes are typically normally distributed with a mean shifted towards superior performance of the right hand. Performance tasks generally require one-to-one assessment and therefore are not time/cost effective.

Hand preference for writing can be easily collected as a tick-box questionnaire leading to large datasets. A recent meta-analysis estimated that the prevalence of left-hand preference in the general population is 10.6% based on data from over 2 million of people [11]. The study also confirmed the effect of some factors influencing hand preference including age, sex, and ethnicity reported in previous research. For example, males are more likely to be left-handed than females [12]; participants of European ancestry have a higher prevalence of left-handedness compared to participants of East Asian ancestry [13].

The etiology and determinants of handedness are still unknown, but many studies have provided evidence of familiar and genetic influence on handedness [14–18]. For example, a child with one left-handed and one right-handed parent is 2 ~ 3 times more likely to be left handed compared with a child with two right-handed parents, and this ratio increases to 3 ~ 4 for a child who has two left-handed parents [15]. Behavior genetic studies have also found that identical twins are more likely to be concordant for hand preference than non-identical or fraternal twins [16]. Medland et al. conducted two large-scale meta-analyses that reviewed a large amount of handedness heritability studies in the literature [17, 18]. Their first study analyzed data from 35 samples of over 21,000 twin pairs from different countries and found that around 25% of the variation in handedness is explained by genetic influences and the rest is explained by environmental factors [17]. Their other study of over 25,000 Australian and Dutch twin families showed again that the genetic influence accounted for around a quarter of variance in hand preference [18].

Less data is available for heritability estimates of handedness indexes or handedness performance. For example, of all the studies analyzed by Medland and colleagues [17], only one used a performance-based measure, i.e. tapping task, and its heritability estimate is nearly zero [19]. In addition, very few studies have looked at the heritability estimates for handedness in populations of non-European ancestry. The prevalence of left-handedness has been consistently reported to be lower in Asia compared to Europe and North America [11, 20, 21]. This difference could be explained by both population-specific genetic and cultural factors. It is clear that stigma against left handedness is an influencing factor which persists in Asia [22–24]. Furthermore, the increasing prevalence of left-handedness with birth year also indicates reduced pressure over time [25, 26]. It is not uncommon to hear how left-handers might have been forced to use the right hand for writing, especially in older generations. Therefore, assessing handedness heritability in younger participants from Asian populations and characterized with measures of both direction and strength might help to better capture the underlying genetic and environmental components.

Left-handedness has been reported to be more prevalent in males and in twins. A large meta-analysis of 144 studies with a total of 1.7 million participants found a sex effect on handedness with a male-to-female odds ratio of 1.27 [12]. The sex difference may be due to the innate biological differences between males and females or culturally transmitted social influences. Apart from this sex effect, an increase of left-handedness prevalence has also been reported in twins in a number of studies [16, 18, 27, 28]. However, other studies have failed to find differences between twins and singletons [29–33]. A complication is that twins and singletons were seldom assessed using the same handedness criteria, recruited in the same manner, or matched for age and sex in these studies.

This study addresses these research questions and gaps in the field by investigating a new sample representative of Hong Kong school-aged children with a set of unique characteristics including: Asian ancestry, a twin and singleton composition, and handedness assessed with different measures, namely the Edinburgh Handedness Inventory and the Pegboard Task. More specifically, we investigated:

1. the distribution of handedness in Chinese school-aged children;
2. sex effects on handedness preference and performance;
3. differences between twins and singletons;
4. heritability estimate of hand preference and performance; and
5. the correlation between different handedness measures and whether they share similar genetic and environmental influences.

## Materials and methods

### Participants

Participants of this study were selected from the Chinese-English Twin Study of Biliteracy, an ongoing longitudinal twin study which focuses on genetic and environmental influences on bilingual development of Chinese children [34]. Twin children from Hong Kong primary schools, with Cantonese as their native language, were recruited for this study. Around four hundred twin pairs have participated since 2015. They were tested on a battery of Chinese language, English language, and cognitive ability tests in the first wave of assessment and were assessed two more times at one-year intervals. Singleton children were also recruited from the same schools as those attended by twins. They were matched with twin participants for age, sex, and grade. The singleton children were assessed once, between the first and the second wave of twin’s data collection.

Handedness data were collected from the twin participants during the second wave of assessment. The sample for the current analysis comprised 410 twin children (or 205 twin pairs) and 426 singleton children. The average age of these twins was 8.7 years (SD = 1.2; age range 6.7 ~ 12.2), and the average age of singletons was 8.3 years (SD = 1.2; age range 6.3 ~ 12.0). The twin sample consisted of 91 monozygotic pairs (41 male pairs and 50 female pairs) and 114 dizygotic pairs (25 male pairs, 21 female pairs, and 68 opposite-sex pairs). Twin zygosity was determined by genotyping the same-sex twins testing small tandem repeat (STR) markers on chromosomes 13, 18, 21, X and Y by Quantitative Fluorescence-Polymerase Chain Reaction (QF-PCR) [35]. The singleton sample consisted of 221 boys and 205 girls. Due to time constraint, 26 twin children and 2 singletons only did the pegboard task but did not respond to the handedness questionnaire; 16 twin children responded to the questionnaire but did not do the pegboard task.

### Measures

#### Handedness questionnaire

Handedness preference was assessed using a modified questionnaire based on the Edinburgh Handedness Inventory (Oldfield, 1971). The questionnaire was translated into Chinese and included 10 items: (1) writing, (2) drawing, (3) throwing, (4) holding scissors, (5) brushing teeth, (6) chopsticks, (7) spoon, (8) knife without fork, (9) broom (upper hand), (10) opening box lid. The full version of the translated questionnaire can be found in Additional file 1. One item in the original Edinburgh inventory, ‘striking a match’, was deemed unsuitable for children [36, 37]. It was replaced by a more culturally relevant item ‘using chopsticks’, which was reported to be learned by Chinese children as early as 5 years old [38]. The Cronbach’s alpha of the 10-item inventory was .852.

The handedness questions were read to a child by a trained research assistant, and the child was asked, for each manual activity, which hand they prefer to use (left hand, right hand or no preference), and then to what degree they use the preferred hand (i.e. always or usually). The self-reported responses were recorded in two columns labeled *Left* and *Right*. If a preference to use a particular hand is ‘Always’, 2 points are put in the preferred column. If a preference is ‘usually’, 1 point is put in the preferred column. If both hands are preferred equally, 1 point is put in both columns. A handedness preference score *EHI* was computed by the formula (RH-LH)/(RH + LH), where RH is the summed score of the right-hand column, and LH is the summed score of the left-hand column. This EHI score ranges from − 1 (all 2 points in the Left column) to + 1 (all 2 points in the Right column). EHI reflects both direction and degree of hand preference: A greater positive score indicates stronger right-handed preference, and a lower negative score indicates stronger left-handed preference.

Handedness direction was determined by a simple transformation of EHI score using a cutoff point. We followed the original criterion set by Oldfield [2] and used the score of zero as a cutoff. A binary variable was created in which children who have a EHI score greater than zero were classified as right-handers and those who have a score less than or equal to zero were classified as left-handers. This new binary variable was named *EHI2*.

From the EHI we extracted the hand preference for ‘writing’ as well as for ‘drawing’, and we recoded each item as a binary of variable with code 1 (right hand preference) and 0 (left hand or no preference). These two items were selected because both tasks require children to use a pen but children may be under different social pressure with regard to their hand choice. A comparison of the two items may indicate a discrepancy of children’s preference for a very similar manual task.

#### PegQ

A continuous handedness index (PegQ) was derived from the pegboard task [1]. The task used a pegboard and 10 dowel pegs, a photo of which can be found in the Additional File 2. A child stood in front of the pegboard and was required to move all the pegs from the furthest to the nearest side as fast as possible using one hand. The time taken to finish moving all 10 pegs was recorded. A practice trial was given before the test. During the formal testing, 5 trials were conducted for each hand, starting with the preferred hand for writing and then alternating between each trial. For each hand, the three best trials out of five were averaged and used in calculating PegQ, formulated as the time difference between the left hand mean (L) and the right hand mean (R), divided by the average time for both hands [2(L-R)/(L + R)]. This PegQ formula has been used in previous studies [39–41]. A positive PegQ score indicates faster or better right-hand and a negative score indicates better left-hand. Similar to what we did for EHI, a handedness performance direction score named *PegQ2*, was converted from PegQ using the score zero as the cutoff point. PegQ2 was coded 1 if PegQ is positive and 0 otherwise.

### Heritability analysis

Six handedness measures were used in the present analysis: writing hand, drawing hand, EHI, EHI2, PegQ and PegQ2. Heritability of different handedness indicators were estimated using a classical twin design in which phenotype variance was partitioned into that due to additive genetic (A), share environmental (C) and non-shared environmental influence (E). The proportions of variance explained by each component of influence were estimated with structural equation modeling and the proportion of variance explained by the genetic influence (A) gives heritability. Using this technique, we fit an ACE model and compared it with its constrained models, such as AE (dropping shared environmental component) or CE model (dropping genetic influence completely).

A univariate ACE model was first fit on the data, separately for each handedness indicators. We first ran a sequence of models to test equality of model parameters by setting them to be equal across different zygosity, twin order, and sex groups. After establishing the homogeneity of all parameters, we tested the full ACE model and compared its fit with various constrained models.

**Fig. 1:**
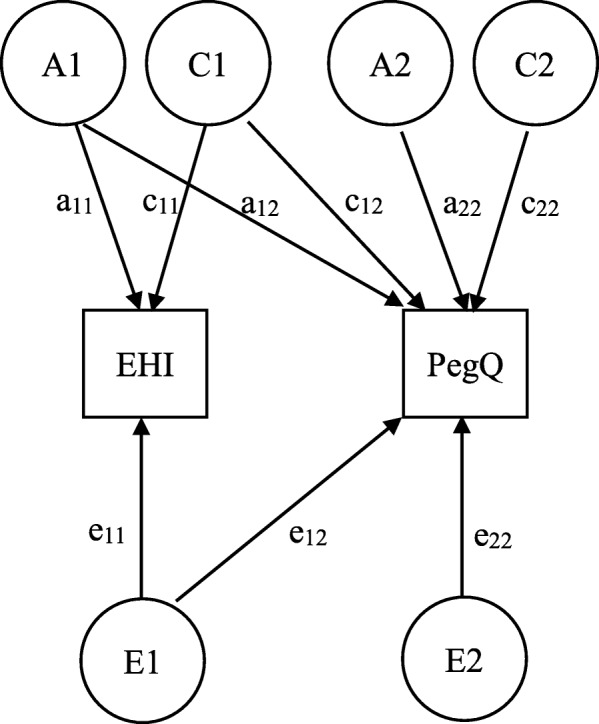
Bivariate Cholesky decomposition of variance and covariance of EHI and PegQ

Next we conducted a bivariate ACE model analysis using the standard Cholesky decomposition approach [42]. The method simultaneously decomposes the variance of two traits into separate variance components which can be represented in a path diagram with six genetic and environmental factors (Fig. 1). The figure shows one set of latent variance components (A1, C1, and E1) is associated with EHI and also with PegQ, whereas another set (A2, C2, and E2) is unique to PegQ only. For each of three sources of influence (A, C, and E), we can estimate three factor loadings which enable us to reconstruct estimates of the contribution of this influence to the variance of EHI, the variance of PegQ, and the covariance between them. Our analysis started with the full ACE bivariate model. Then we tested whether the full model could be modified to a more parsimonious model by dropping some of the parameters.

The univariate and multivariate genetic analyses were performed using the OpenMx software package 2.12.2 [43]. The program to estimate heritability was adapted from the OpenMX scripts distributed on the International Workshop on Statistical Genetic Methods for Human Complex Traits [44]. All analyses scripts are available through Open Science Framework https://osf.io/pcg8m/.

## Results

### Sex and twin effects on handedness preference

A total of 384 twin children and 424 singletons responded to the Edinburgh Handedness Inventory. For each item listed in the inventory, an item-total correlation was first calculated (Table 1). The two items with the highest item-total correlations are ‘writing hand’ (.72) and ‘drawing hand’ (.72), and the two least correlated items are ‘using a broom’ (.36) and ‘opening a box lid’ (.41). Next, the raw response of each item were reorganized into three categories: 1) left-hand preference (‘always left’ & ‘usually left’), 2) no preference, and 3) right-hand preference (‘always right’ & ‘usually right’). Data were split by sample (twin vs. singleton) and by sex. Proportion of each hand preference group was compared between sex in the twins and singletons respectively, and then between twins and singletons overall. A 3 × 2 chi-square contingency test was conducted for each item and 10 comparisons were made in each split data group. The significant level of the approximated Bonferroni’s correction for multiples tests was calculated as *p* = .05/10 = .005.

**Table 1:** Edinburgh Handedness Inventory data split by sample and sex

	Item	Preference	Twins			Singletons			Total		
Item	total		Male	Female	χ^2^_(df = 2)_	Male	Female	χ^2^_(df = 2)_	Twins	Singletons	χ^2^_(df = 2)_
	*r*	Class	*N* = 187	*N* = 197		*N* = 220	*N* = 204		*N* = 384	*N* = 424	
		LH	13 (7.0%)	14 (7.1%)		16 (7.3%)	9 (4.4%)		27 (7.0%)	25 (5.9%)	
1. Writing	.717	NP	4 (2.1%)	0 (0.0%)	4.26	3 (1.4%)	5 (2.5%)	2.17	4 (1.0%)	8 (1.9%)	1.37
		RH	170 (90.9%)	183 (92.9%)		201 (91.4%)	190 (93.1%)		353 (91.9%)	391 (92.2%)	
		LH	15 (8.0%)	13 (6.6%)		16 (7.3%)	12 (5.9%)		28 (7.3%)	28 (6.6%)	
2. Drawing	.718	NP	11 (5.9%)	8 (4.1%)	1.02	11 (5.0%)	17 (8.3%)	2.14	19 (4.9%)	28 (6.6%)	1.11
		RH	161 (86.1%)	176 (89.3%)		193 (87.7%)	175 (85.8%)		337 (87.8%)	368 (86.8%)	
		LH	16 (8.6%)	20 (10.2%)		30 (13.6%)	13 (6.4%)		36 (9.4%)	43 (10.1%)	
3. Throwing	.427	NP	65 (34.8%)	69 (35.0%)	0.32	99 (45.0%)	83 (40.7%)	8.99*	134 (34.9%)	182 (42.9%)	6.49*
		RH	106 (56.7%)	108 (54.8%)		91 (41.4%)	108 (52.9%)		214 (55.7%)	199 (46.9%)	
		LH	16 (8.6%)	12 (6.1%)		23 (10.5%)	19 (9.3%)		28 (7.3%)	42 (9.9%)	
4. Holding Scissors	.659	NP	25 (13.4%)	18 (9.1%)	2.86	43 (19.5%)	20 (9.8%)	8.57*	43 (11.2%)	63 (14.9%)	4.66
		RH	146 (78.1%)	167 (84.8%)		154 (70.0%)	165 (80.9%)		313 (81.5%)	319 (75.2%)	
		LH	16 (8.6%)	12 (6.1%)		26 (11.8%)	12 (5.9%)		28 (7.3%)	38 (9.0%)	
5. Brushing Teeth	.585	NP	44 (23.5%)	49 (24.9%)	0.89	57 (25.9%)	47 (23.0%)	5.75	93 (24.2%)	104 (24.5%)	0.81
		RH	127 (67.9%)	136 (69.0%)		137 (62.3%)	145 (71.1%)		263 (68.5%)	282 (66.5%)	
		LH	11 (5.9%)	14 (7.1%)		20 (9.1%)	14 (6.9%)		25 (6.5%)	34 (8.0%)	
6. Chopsticks	.689	NP	8 (4.3%)	2 (1.0%)	4.19	9 (4.1%)	8 (3.9%)	0.73	10 (2.6%)	17 (4.0%)	2.01
		RH	168 (89.8%)	181 (91.9%)		191 (86.8%)	182 (89.2%)		349 (90.9%)	373 (88.0%)	
		LH	15 (8.4%)	17 (9.0%)		20 (9.1%)	12 (5.9%)		32 (8.7%)	32 (7.5%)	
7. Spoon	.559	NP	41 (23.0%)	34 (18.1%)	1.38	57 (25.9%)	66 (32.4%)	3.13	75 (20.5%)	123 (29.0%)	7.61*
		RH	122 (68.5%)	137 (72.9%)		143 (65.0%)	126 (61.8%)		259 (70.8%)	269 (63.4%)	
		LH	17 (9.6%)	18 (9.6%)		26 (11.9%)	32 (15.8%)	4.53	35 (9.6%)	58 (13.8%)	
8. Knife without fork	.520	NP	24 (13.6%)	14 (8.0%)	3.02	45 (20.5%)	27 (13.4%)		39 (10.7%)	72 (17.1%)	11.57**
		RH	136 (76.8%)	155 (82.4%)		148 (67.6%)	143 (70.8%)		291 (79.7%)	291 (69.1%)	
		LH	30 (16.9%)	26 (13.9%)		40 (18.2%)	30 (14.7%)		56 (15.3%)	70 (16.5%)	
9. Broom	.362	NP	49 (27.5%)	42 (22.5%)	2.44	61 (27.7%)	51 (25.0%)	1.79	91 (24.9%)	112 (26.4%)	0.57
(Upper hand)		RH	99 (55.6%)	119 (63.6%)		119 (54.1%)	123 (60.3%)		218 (59.7%)	242 (57.1%)	
		LR	11 (6.2%)	20 (10.7%)		20 (9.1%)	21 (10.3%)		31 (8.5%)	41 (9.7%)	
10. Opening a box lid	.410	NP	78 (43.8%)	69 (36.9%)	3.38	116 (52.7%)	95 (46.6%)	1.61	147 (40.3%)	211 (49.8%)	9.10*
		RH	89 (50.0%)	98 (52.4%)		84 (38.2%)	88 (43.1%)		187 (51.2%)	172 (40.6%)	

1) Q6 ‘chopsticks’ is a modified item to replace ‘striking a match’ in the original Edinburgh Handedness Inventory

2) For each task responses were recoded into three categories: ‘LH’, ‘NP’, and ‘RH’, in which LH = Left-handed. NP=No preference, and RH = Right-handed

3) Due to time constraint, 26 twin children and 2 singletons did not respond to the handedness questionnaire but participated in other tasks

4) Group difference for each item was compared by the 3 × 2 χ^2^ test and the significance was marked by * *p* < .05, and ** *p* < .005. *p* = .005 is the significant level after Bonferroni correction for 10 comparisons

The writing item in the EHI showed that there were slightly more left-handers in the twin sample (7.0%) than in the singleton sample (5.9%). When ‘no-preference’ was merged with left hand preference for writing, the proportions of non-right-handedness in the two samples were about equal at 8%. For the drawing hand, there were also slightly more left-handers (7.3%) in the twins than in the singletons (6.6%). If considering children without a preference, the frequency of non-right-handedness increased to 12.2% in the twins and 13.2% in the singletons, respectively. A cross-tabulation analysis of the two items showed that the discrepancy between writing and drawing resulted mainly from a number of participants who wrote with the right hand but had no preference when they draw (5.0% of the total sample, comprising of 17 twin children and 23 singletons). There were another 0.9% participants (2 twin children and 5 singletons) who reported writing with the right but drew with the left hand.

Change of the preferred hand for writing was often reported in Chinese children [45]. Although information about handedness switch was not asked directly, it is possible to estimate the number of children who might have been encouraged to change the writing hand. We calculated a new composite score using the eight activities other than writing or drawing, and compared this score with the writing task. We found that 2.2% children (4 twins and 14 singletons) reported writing with the right hand but showed an overall preference for the left hand (i.e., their summed score of the left-handed items excluding writing/drawing was greater than the right-handed sum). We speculated that these children might have experienced a forced handedness switch in their early childhood.

A comparison between twins and singletons yielded no convincing evidence of increased left-handedness in twins than in singletons as reported in some studies [46], despite the observation that % of left-handed ‘writers’ is slightly higher in twins than in singletons (7.0% vs 5.9%). Contrary to our expectation, some tasks revealed a higher prevalence of left-handers among singletons as compared to twins. For example, there were 13.8% singletons vs. 9.6% of twins ‘using a knife’ with their left-hand. When comparing males and females, our results are in line with previous reports that males are more prone to left-handedness [12]. There were 7.3% boys in the singleton sample who reported ‘writing’ with the left-hand, in contrast to only 4.4% girls who had the same behavior. Two other items (‘throwing’ and ‘holding scissors’) showed girls’ greater tendency toward right-handedness. There were more girls who prefer to use their right hand than boys when they ‘throw’ (52.9% vs. 41.4%) or when they ‘hold scissors’ (80.9% vs. 70.0%). However, neither comparison reached a statistically significant level after Bonferroni’s correction, and neither result could be replicated in the twin sample.

Next we compared the proportions of two handedness direction groups in the twins and singletons based on a binary classification of writing hand and drawing hand, as well as on the derived EHI2 and PegQ2 measures (Table 2). Overall our analysis found no significant differences and do not support previous findings suggesting a higher prevalence of left-handedness in twins compared to singletons [47, 48]. In fact, we observed the opposite trend for some measures: 12.2% vs. 13.2% for the preferred hand for drawing, 6.5% vs. 8.5% for EHI2, and 13.2% vs. 15.3% for PegQ2. Instead, consistent with previous literature, we found that left- handers were more prevalent in males than females for both writing hand preference and EHI2, in both twins and singletons. The PegQ2 measure also detected a higher proportion of left-handed males compared to females but in the singleton sample only (18.1% vs. 12.2%).

**Table 2:** Number and percent of non-right-handers (NRH) and right handers (RH) by sample and sex for four handedness direction indicators

	Twins			Singletons			Total		
Handedness Direction	Male n (%)	Female n (%)	χ^2^_(*df* = 1)_	Male n (%)	Female n (%)	χ^2^_(*df* = 1)_	Twins n (%)	Singletons n (%)	χ^2^_(*df* = 1)_
Writing hand									
NRH	17 (9.1%)	14 (7.1%)	0.51	19 (8.6%)	14 (6.9%)	0.46	31 (8.1%)	33 (7.8%)	0.02
RH	170 (90.9%)	183 (92.9%)		201 (91.4%)	190 (93.1%)		353 (91.9%)	391 (92.2%)	
Drawing hand									
NRH	26 (13.9%)	21 (10.7%)	0.94	27 (12.3%)	29 (14.2%)	0.35	47 (12.2%)	56 (13.2%)	0.17
RH	161 (86.1%)	176 (89.3%)		193 (87.7%)	175 (85.8%)		337 (87.8%)	368 (86.8%)	
EHI2									
NRH	13 (7.0%)	12 (6.1%)	0.12	20 (9.1%)	16 (7.8%)	0.21	25 (6.5%)	36 (8.5%)	1.13
RH	174 (93.0%)	185 (93.9%)		200 (90.9%)	188 (92.2%)		359 (93.5%)	388 (91.5%)	
PegQ2									
NRH	23 (12.1%)	29 (14.2%)	0.38	40 (18.1%)	25 (12.2%)	2.87	52 (13.2%)	65 (15.3%)	0.71
RH	167 (87.9%)	175 (85.8%)		181 (81.9%)	180 (87.8%)		342 (86.8%)	361 (84.7%)	

1) For the binary classification of ‘writing hand’ and ‘drawing hand’, NRH includes both left-hand preference and no preference

2) EHI2 and PegQ2 are the binary classification of EHI and PegQ scores respectively, using zero as the cutoff point. NRH refers to those who scored less or equal to zero. There are 4 participants (including 1 twin and 3 singletons) who scored exactly zero on EHI

We then compared quantitative scores of handedness, measured as EHI and PegQ, between twins and singletons and between males and females (Table 3). The frequency distribution graph of the two scores can be found in Additional file 3. The EHI mean was .641 (SD = .415) in the twins and .577 (SD = .393) in the singletons, respectively (Table 3). The mean PegQ score was .094 (SD = .101) in the twins and .097 (SD = .100) in the singletons. A sample by sex (2 × 2) ANOVA shows that EHI was significantly higher in twins than in singletons (*F*_1,804_ = 4.84, *p* = .03), suggesting twins have a stronger right-hand preference than singletons. Furthermore, EHI was marginally higher in females than in males (*F*_1,804_ = 3.73, *p* = .07), indicating that males have a tendency towards left-hand preference. There was no interaction effect between sample and sex (*F*_1,804_ = 0.07, *p* = .79). With regard to the comparison of PegQ, the difference between twins and singletons was negligible (*F*_1,816_ = 0.11, *p* = .74). Neither was there a sex difference (*F*_1,816_ = 0.38, *p* = .54), nor an interaction effect (*F*_1,816_ = 0.03, *p* = .86).

**Table 3:** Mean EHI and PegQ score by sample and sex

Handedness	Twins		Singletons		Total	
Score	Male	Female	Male	Female	Twins	Singletons
EHI						
N	187	197	220	204	384	424
Mean	.618	.662	.548	.607	.641	.577
SD	.422	.410	.427	.351	.416	.393
PegQ						
N	190	204	221	205	394	426
Mean	.093	.096	.094	.099	.094	.097
SD	.097	.105	.106	.093	.101	.100

### Correlation between different handedness measures

Correlations across laterality measures and their association with age and sex show similar patterns in the twin and in the singleton sample (Table 4). PegQ was moderately correlated (about .40) with both preferred hand for writing and EHI in both samples. A similar correlation was found between their binary classification scores, EHI2 and PegQ2. Neither EHI nor PegQ was correlated with age and sex.

**Table 4:** Correlations between age, sex, and different measures of handedness in the twin sample (lower triangle) and in the singleton sample (upper triangle)

	Age	Sex	Writing hand	Drawing hand	EHI	EHI2	PegQ	PegQ2
Age		.05	.02	.04	.07	.08	−.06	.02
Sex	.01		.03	−.03	.08	.02	.03	.08
Writing hand^a^	.00	.04		.62**	.69**	.73**	.40**	.34**
Drawing hand ^a^	−.03	.05	.71**		.67**	.66**	.29**	.24**
EHI	−.02	.05	.80**	.72**		.81**	.42**	.35**
EHI2^a^	−.07	.02	.85**	.71**	.84**		.44**	.39**
PegQ	.08	.02	.44**	.37**	.44**	.44**		.70**
PegQ2 ^a^	.08	−.03	.46**	.34**	.43**	.44**	.67**	

Measures marked with ^a^ are binary handedness variables with 0 = NRH and 1 = RH;

Sex is coded as 0 = male, 1 = female; * *p* < .05, ** *p* < .01

### Univariate heritability analysis

We conducted a series of univariate genetic analyses to estimate sources of variation in handedness that is due to genetic and environmental influence (Table 5). We compared the full ACE model with its constrained sub-models AE, CE and E only model and tested their differences in fit statistics.

**Table 5:** Univariate ACE model fitting results for different handedness measures and estimates of variance components of A, C, and E

Measure	*%* Variance			Fit of ACE Model		Fit of Constrained Models			*ΔLL* (vs. ACE Model)		
	*A*	*C*	*E*	*-2LL*	*df*	*AE*	*CE*	*E*	*AE*	*CE*	*E*
Writing hand ^a^	.266	.000	.734	213.69	379	213.69	214.36	214.64	0.00	0.67	0.94
Drawing hand^a^	.235	.000	.765	281.75	379	281.75	282.59	282.93	0.00	0.84	1.18
EHI	.205	.000	.795	3945.86	380	3945.86	3951.25	3951.25	0.00	5.39*	5.39
EHI2 ^a^	.376	.000	.624	178.66	379	178.66	179.55	180.44	0.00	0.89	1.78
PegQ	.217	.000	.783	2934.79	390	2934.79	2937.02	2939.54	0.00	2.23	4.75
PegQ2^a^	.253	.000	.747	302.96	389	302.96	302.65	304.34	0.00	0.69	1.38

Measures marked with ^a^ are binary handedness variables. *p < .05

Heritability or genetic contribution (proportion of A) to various handedness measures was generally low. The highest heritability estimate was found for EHI2 (.38), which was the binary classification of handedness based on overall preference score EHI. However, the estimate was not significant, meaning we cannot exclude an environmental explanation with CE model or E only model. For all other handedness indicators, heritability estimates were consistently in the range of .20 to .30. The heritability for EHI in particular was .21 and a model without genetic component (CE) fit significantly worse than the full ACE model, indicating that the genetic contribution to hand preference is weak but significant.

### Bivariate heritability analysis

EHI and PegQ had a correlation of about .40 (Table 4). Through a bivariate genetic analysis using the Cholesky decomposition method, we asked whether this correlation might result from shared genetic influences. The bivariate analysis was performed for only EHI and PegQ, because they are continuously distributed, giving us more power to detect additive genetic effect than the categorical measures in our study (Table 6).

**Table 6:** Bivariate Cholesky model fitting results for EHI and PegQ and comparisons of nested models

	ACE and nested models	*-2LL*	*df*	*AIC*	*ΔLL*	*Δdf*	*p*
1	Model ACE	6796.36	767	5262.36			
2	Model AE, drop C	6801.79	770	5261.79	5.43	3	.14
**2a**	**Model AE1, drop*a***_***12***_ (a_12_ = 0)	**6802.73**	**771**	**5260.73**	**6.37**	**4**	**.17**
2b	Model AE2, drop a_22_ (a_22_ = 0)	6806.98	771	5264.98	10.62	4	.03
3	Model CE, drop A	6806.58	770	5266.58	10.22	3	.02
4	Model E, drop A & C	6813.13	773	5267.13	16.78	6	.01

The best fitted model is highlighted with the bold font

The full ACE model fit result was tested first (Model 1). Next, we tested whether shared environmental effect, or the two C factors, could be completely dropped (Model 2 or AE). Dropping C did not result in a significant loss in likelihood function statistics (−2LL); therefore, Model 2 was accepted as more parsimonious than the full ACE model. This is consistent with what we have observed in the univariate analyses which found little shared environmental influences on either EHI or PegQ. Furthermore, we tested two reduced models of AE. In Model 2a, we dropped the correlation between the two genetic factors by constraining the parameter a_12_ to be zero. This did not lead to a significant change in fit statistics, indicating the genetic correlation may be dropped and the two genetic factors A1 and A2 are independent. In Model 2b, we tested an alternative model in which a_12_ was kept free but a_22_ was fixed to zero. This is equivalent to dropping A2 from the model, meaning EHI and PegQ share the same genetic influence. However, dropping A2 worsened the fit significantly and thus Model 2b was rejected, implying that sources of genetic influences of EHI and PegQ are not identical. Lastly, we tested two environmental models, the CE model (dropping A completely) and the E only model (dropping both A and C). Fitting both models resulted in a significant reduction in model fit statistics, indicating that a pure environmental explanation of handedness cannot not be accepted.

**Fig. 2:**
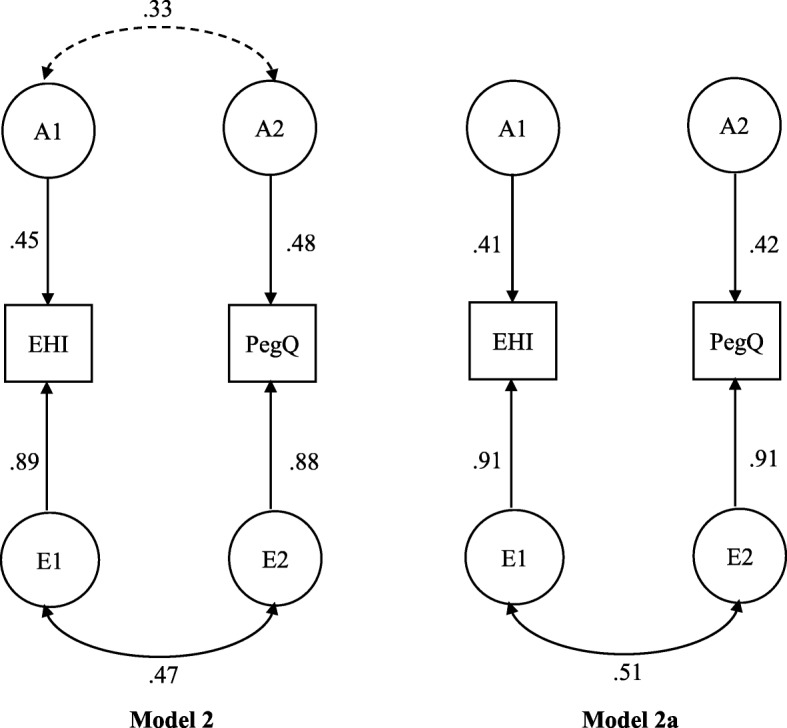
Standardized parameter estimates for the bivariate AE model (Model 2) and its reduced best-fitted model (Model 2a). Dashed line indicates non-significant correlation

The above analyses show that a constrained AE model without genetic correlation (Model 2a) explains the variance and covariance of the two handedness measures as well as the full ACE model, but with many fewer parameters. Different models can also be compared using the Akaike information criteria (AIC), a popular model fit index. The model with the lowest value of AIC reflects the best balance of goodness of fit and parsimony. Therefore, Model 2a was accepted as the best bivariate model. For ease of result interpretation, we converted the Cholesky model to an equivalent correlated factor model in which the association between latent factors are modelled explicitly as factor correlations, as recommended by some authors [49]. Figure 2 shows model AE (Model 2) and its constrained model (Model 2a) in this representation and all the standardized parameter estimates. The figure shows that variation in EHI and PegQ are influenced by two different additive genetic factors (A1 and A2) and two different environmental factors (E1 and E2). The association between A1 and A2 was non-significant, indicating little overlap between genetic influences of EHI and PegQ. The observed association between EHI and PegQ arose entirely from the correlation of the two environmental factors based on the best fitted Model 2a.

## Discussion

In this study, we investigated handedness data in over 800 Chinese children using different binary and continuous measures. Our results show that, using a binary classification of writing hand as the criterion, around 8% of school-aged children are left-handers. This estimate is lower than the most recently published value of 10.6% for the general left-handedness prevalence [11], but still higher than what was previously reported in Chinese. A 1980s survey found that less than 1% Chinese students were left-handed writers [24]. However, those data refer to the mainland Chinese population and were collected more than 30 years ago, whereas our study was conducted in Hong Kong, a city culturally influenced by the West. Large-scale population studies in North America and Britain have shown that the prevalence of left-handers varies by birth year and age cohort, and frequency of left-handers is much higher in the younger generations [25, 26]. In Hong Kong, left-handers used to face strong pressure to conform with the majority forced to switch their writing hand at school [45], but our data suggest that the pressure in enforcing the use of the right hand for writing has reduced in recent decades. Although we did not have direct information on possible hand switching experience of our participants, we could infer this information from the difference between writing and other unimanual activities. Around 6% of the children in our sample reported writing with the right hand but drawing with the left hand or with no preference. We also found around 2% participants who were right-handed writers but showed an overall preference for the left hand across items. Some of these children might have been pressured to change the writing hand, but they might also have opted to do so themselves either due to weak hand preference or due to the observation of peers and adults around them who are mostly right-handed. Considering these different reasons for inconsistent hand use, our estimation of children who experienced forced handedness switch is around 2%. Including this possible handedness switch group, we can state that the incidence rate of left-handedness in the Chinese children is comparable with the worldwide average.

The proportion of left-handers derived from the overall handedness preference EHI2 was also around 8% (7% in twins and 9% in singletons). Instead, a recent study conducted in mainland China, which collected EHI data from over 1800 college students, found only less than 4% left-handers [50]. It has been reported that the pressure against left-hand use still persists in mainland China and there remain discriminatory education practices against left-hander in schools [51]. Considering large geographical and environmental variation, more data are needed to determine the prevalence of left-handedness in the general Chinese population and to track the possible change of attitude and adaptation to handedness diversity over time. Our data suggest that the increase of left-handedness prevalence in Hong Kong in recent times is likely to reflect effects of Westernization. Therefore, it is reasonable to predict that the lower proportion of left-handedness in East-Asian populations is more likely to result from cultural rather than genetic factors.

We also calculated prevalence of left-handedness separately in twins and singletons. We found no evidence that left-handedness is more common in twins as suggested in some previous studies [16, 28]. We did observe a slightly higher frequency of left-handed writer in twins than singletons (7% vs. 6%), but the difference was not significant and nearly non-existent if ‘no preference’ respondents were taken into account. In contrast, when we defined left-handedness by the overall handedness preference (EHI2), we found an opposite trend that left-handers were more frequent in singletons (9%) than twins (7%). The difference was particularly pronounced when we compared the quantitative score EHI in a multivariate analysis controlling for the age and sex effect, which showed that singletons, rather than twins, have a stronger degree of left-handedness. More left-handers in singletons were also observed when handedness was defined by pegboard performance (PegQ2). There were 15% singleton children who were better with their left hand compared to 13% in twins, though the difference did not reach statistical significance. Nevertheless, our study contradicted the previous finding that left-handers are more common in twins compared to singletons. The reason for the inconsistent findings might be due to using the overall preference index (EHI) rather than a single indicator of writing hand to define handedness. Our item cross-tabulation analysis showed that a handedness difference between twins and singletons mainly arose from tasks such as throwing, cutlery use, and opening a box etc. It is unclear why there were distinct rates of no-preference respondents and left-handers between them. More research is needed to understand this behavior discrepancy.

We point out that we adopted a dichotomous classification of handedness in EHI2 and PegQ2 using zero as the cutoff value, which might be suboptimal compared to a three-group classification scheme. Only 4 children (1 twin and 3 singletons) in our sample had scored exactly zero. The number is too small to justify a separate category. Although alternative cutoff points have been empirically validated and recommend for handedness classification [4], there remains no consensus regarding the best classification method among researchers who use the EHI [52]. Furthermore, one of the purposes of this study is to compare prevalence of handedness based on preference and performance measures, and a consistent classification criterion, i.e. using zero as the cutoff score, is important for this comparison.

Our data support the widely reported finding that there is a higher incidence of left-handedness among males than females [12]. There were 9% of boys in our sample who were left-handed or ambidextrous writers as compared to only 7% of girls (Table 2). Male children also showed stronger degree of left-handed preference as reflected by their lower average EHI score (Table 3). The individual preference item analysis showed that the sex difference was particularly pronounced in the singleton sample. Our results also found a discrepancy between boys and girls in the singleton sample on their pegboard performance scores, continuous or categorical. Though our sample is too small for the detection of significant sex effect, the pattern is consistent with a recent study in UK which found females tend to be more right lateralized and males are more left lateralized for PegQ [53].

In this study we are trying to estimate the heritability of hand preference in a population from Hong Kong and the heritability of a continuous measure of handedness in the same population. Our heritability estimates for various handedness measures are consistent with previous studies. We found that heritability for writing hand preference is .27 (Table 5). It has been well established in previous meta-analyses that around a quarter of handedness variation is explained by additive genetic effect and the remaining is purely non-shared environmental influences [17, 18]. However, the phenotypes used in those studies were predominantly preference measures, especially writing hand, and the samples collected were mostly from North America or Europe. Our heritability estimates for different categorical measures converted from EHI and PegQ ranged from .24 to .38 with the highest figure observed for EHI direction or EHI2. Due to relatively small sample sizes, we could not rule out alternative or environmental explanations of data for these categorical measures.

Our heritability estimates for the two quantitative scores of preference (EHI) and performance (PegQ) were .21 and .22, respectively. These modest values do not support the idea that quantitative traits might have a higher heritability than categorical data, as reported in a previous study [54]. One difficulty in comparing our results with this study is the age of participants as we used school-aged children instead of adults. There is evidence that strength of hand preference becomes stronger with age [55, 56] and handedness preference might have not been fully established in our samples. Performance measures are also expected to change with age for exposure to daily activities such as writing, throwing, and other unimanual skills reinforcing the use of the preferred hand [57]. More studies with a wider age range are needed to reach consensus on heritability estimates for different handedness measures.

The PegQ quantitative measure identified 14 ~ 15% children who performed faster with their left hand on the pegboard. Around 2/3 of them also had a left-hand preference by EHI direction. This is consistent with the overall correlation of .40 ~ .50, indicating that the two measures do not map exactly on to each other. These results also concur with the aforementioned recent study conducted on UK children showing poor correlations across different handedness measures [53]. Our bivariate genetic modelling analysis further revealed that a model with one latent genetic factor did not fit the data. The overlap between genetic influence on handedness preference and performance is only moderate and not statistically significant. Based on our findings, an important implication for future research, especially in molecular genetic studies, is that preference and performance represent two different handedness definitions, and it is important not to use them as interchangeable measures. Different measures will underpin different modelling of genetic association studies [58].

There are two major limitations of our study. First, with a sample of only ~ 200 twin pairs, the power to detect a genetic correlation of .50 between phenotypes with a heritability of .20 ~ .30 was reported to be only around .50 or less [59]. Therefore, although our model fitting results show that the independent genetic factor model (Model 2a) fits the data best, we could not rule out a possible moderate association between their genetic origin due to low power. The small sample also limited our ability to further compare handedness between zygosity groups. For example, girls with a male co-twin have a higher EHI (.706 vs. .675) and also a higher PegQ (.091 vs. .088) than girls with a female co-twin. This is consistent with previous findings showing that having a male co-twin increased the probability of right-handedness in females [60]. However, with our sample of only 21 female pairs and 68 opposite-sex pairs, the differences are too small to be conclusive.

Second, some of the items on the EHI might have been affected by the age of study participants. We replaced one of the original items ‘striking a match’ with ‘using chopsticks’ to adjust the inventory for children, but some other items could have been problematic. For instance, ‘using a broom’ and ‘opening a box lid’ have been suggested to be subjected to ambiguous interpretations [61]. Our results show that these two tasks have the lowest item-total correlation among all items on the inventory. Furthermore, we used only one laterality index (the PegQ), and it should be noted that handedness performance is not a one-dimensional trait, but rather a multi-dimensional one that includes different aspects such as dexterity, skill, and strength [53]. There has been evidence that using a single hand performance does not always correctly classify an individual who has a left-or right-handed preference, and a combination of different measures might be more effective [62].

## Conclusions

In summary, we report on a novel set of data from an underrepresented population which allowed us to address questions in the field of laterality research. Our results reinforce the idea that different handedness measures tap into different laterality dimensions and provide a reference dataset for studies in Asian populations.

## Supplementary information

**Additional file 1.** The Chinese translated Edinburgh Handedness Inventory.

**Additional file 2.** The pegboard and dowel pegs.

**Additional file 3.** Distribution of EHI and PegQ in the twin and in the singleton sample. All files have been uploaded to the Open Science Framework repository (https://osf.io/pcg8m/).

## Data Availability

The datasets analyzed during the current study are available from the corresponding author upon reasonable request. The code for the heritability analysis is available at https://osf.io/pcg8m/.

## Abbreviations

EHI: Edinburgh Handedness Inventory score
EHI2: Direction of handedness preference based on EHI using 0 as a cutoff
PegQ: Pegboard laterality quotient
PegQ2: Direction of handedness performance based on PegQ using 0 as a cutoff
LH: Left-handed
RH: Right-handed
NRH: Non-right-handed
NP: No hand preference
