# Supplementary material for: Prevalence and heritability of handedness in a Hong Kong Chinese twin and singleton sample
Source: BMC Psychol. 2020 Apr 22;8:37. doi: 10.1186/s40359-020-00401-9 (PMC7178737; doi:10.1186/s40359-020-00401-9)
Supplement: Supplementary file 1 — Additional file 1. The Chinese translated Edinburgh Handedness Inventory. [file 40359_2020_401_MOESM1_ESM.docx]

**The Chinese Version of the Edinburgh Handedness Inventory Used in Current Study**

提問：「我陣間會問你平時用邊隻手做依啲動作。你可以答我左手，右手或者左右手都得。」

Instruction: I am going to ask you which hand you usually use to do the following actions. You can say left hand, right hand, or both hands.

若學生回答常用 左手/右手時，請接著問: 「係一定要用呢隻手 定係 另外嗰隻手都得不過用得無咁叻？」

Follow up: If the child answers left/right hand, please follow up by asking: “Do you only use this hand, or can you also use the other hand, though a bit clumsily?

評分方法：

若學生回答**左右手都得**,請給左手和右手各1分。

Scoring: if the child uses **both hands**, please give 1 mark to each hand.

若學生回答**一定要用**左/右手，請給慣用的那隻手2分，不用的那隻手0分。

If the child uses **only left/right hand**, please give 2 marks to the hand usually used, and 0 mark the hand not used.

若學生回答**另外嗰隻手都得**，請給較好的那隻手1分，較差的那隻手0分。

If the child can also **use the other hand but a bit clumsily**, please give 1 mark to the preferred hand, and 0 mark to the hand not preferred.

| 項目 Activity | 左手 (Left Hand) | 右手 (Right Hand) |
| --- | --- | --- |
| 1. 寫字 Writing |  |  |
| 1. 畫畫 Drawing |  |  |
| 1. 擲東西 Throwing |  |  |
| 1. 用剪刀 Holding Scissors |  |  |
| 1. 刷牙 Brushing Teeth |  |  |
| 1. 筷子 Chopsticks * |  |  |
| 1. 匙羹 Spoon |  |  |
| 1. 拿刀 (無叉) Knife without fork |  |  |
| 1. 掃把 (上面那隻手) Broom (Upper hand) |  |  |
| 1. 打開盒蓋 Opening a Box (Lid) |  |  |
| **Summed Score of 1 - 10** | LH | RH |
| EHI Hand Preference Degree = | $\frac{(RH-LH)}{(RH+LH)} \times100$ | |
