# Supplementary figures and images for: Prevalence and heritability of handedness in a Hong Kong Chinese twin and singleton sample
Source: BMC Psychol. 2020 Apr 22;8:37. doi: 10.1186/s40359-020-00401-9 (PMC7178737; doi:10.1186/s40359-020-00401-9)

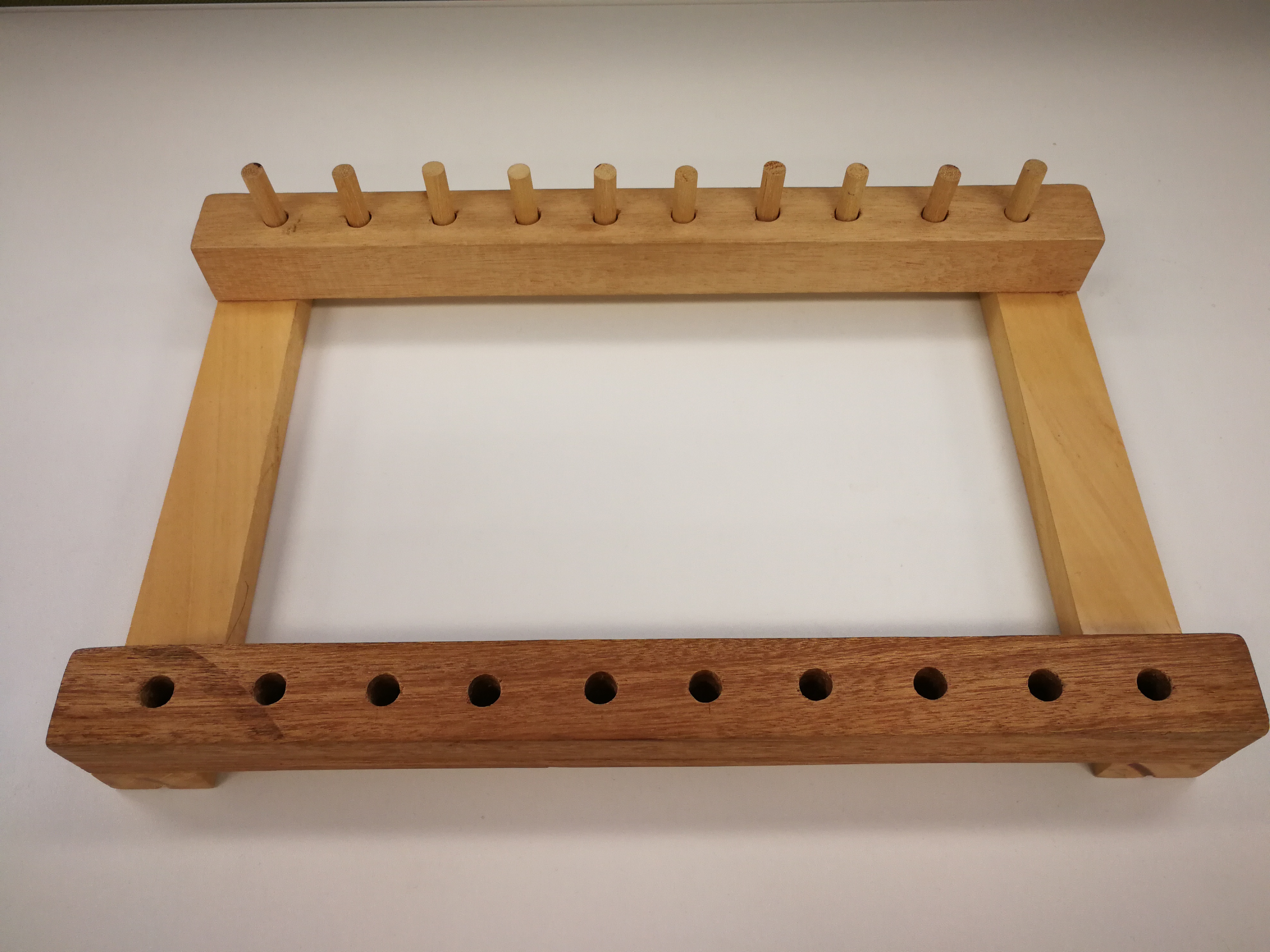

Supplement: Supplementary file 2 — Additional file 2. The pegboard and dowel pegs. [file 40359_2020_401_MOESM2_ESM.jpg]
