# Supplementary material for: Prevalence and heritability of handedness in a Hong Kong Chinese twin and singleton sample
Source: BMC Psychol. 2020 Apr 22;8:37. doi: 10.1186/s40359-020-00401-9 (PMC7178737; doi:10.1186/s40359-020-00401-9)
Supplement: Supplementary file 3 — Additional file 3. Distribution of EHI and PegQ in the twin and in the singleton sample. All files have been uploaded to the Open Science Framework repository (https://osf.io/pcg8m/). [file 40359_2020_401_MOESM3_ESM.docx]

**Distribution of EHI and PegQ score in the twin and in the singleton sample**

| Twins | Singletons |
| --- | --- |
| 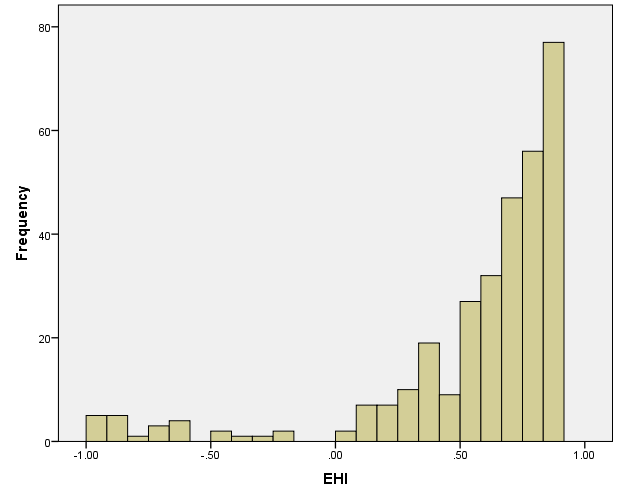 | 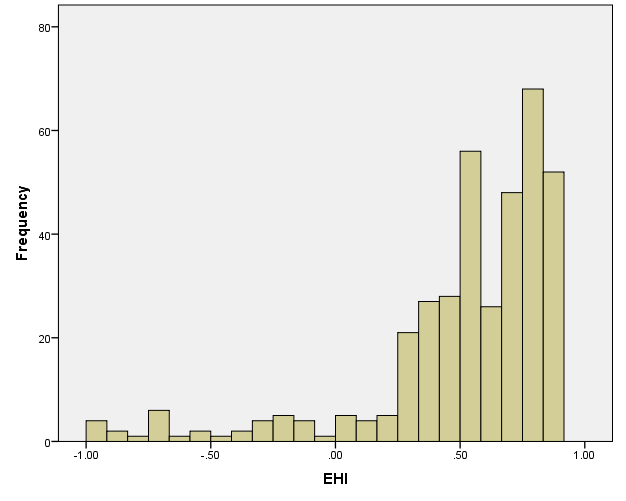 |
|  |  |
| Twins | Singletons |
| 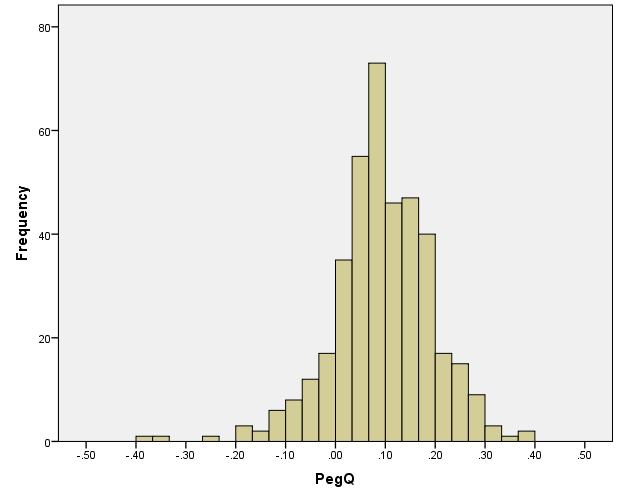 | 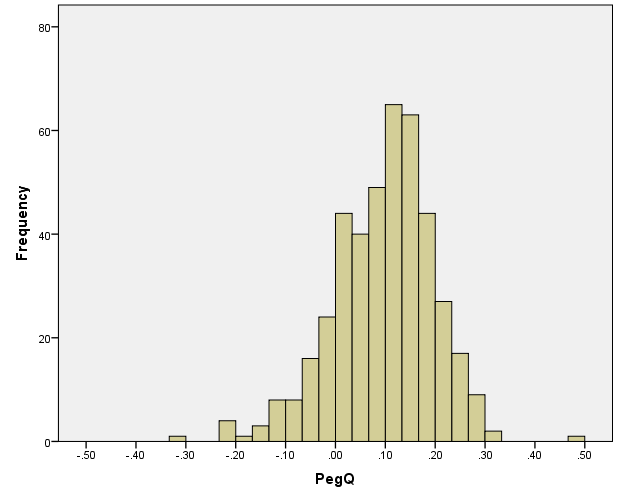 |
